# The Influence of Silica Nanoparticles on the Thermal and Mechanical Properties of Crosslinked Hybrid Composites

**DOI:** 10.3390/ma14237431

**Published:** 2021-12-03

**Authors:** Tomasz Klepka, Beata Podkościelna, Dariusz Czerwiński, Bronisław Samujło

**Affiliations:** 1 Department of Technology and Polymer Processing, Faculty of Mechanical Engineering, Lublin University of Technology, Nadbystrzycka 36, 20-618 Lublin, Poland; b.samujlo@pollub.pl; 2 Department of Polymer Chemistry, Institute of Chemical Science, Faculty of Chemistry, Maria Curie-Skłodowska University, M. Curie-Skłodowska Sq. 3, 20-031 Lublin, Poland; beata.podkoscielna@mail.umcs.pl; 3 Department of Computer Science, Lublin University of Technology, Nadbystrzycka 36, 20-618 Lublin, Poland; d.czerwinski@pollub.pl

**Keywords:** silica, nanoparticles, hybrid composites, mechanical properties, Fréchet’s theory

## Abstract

This paper presents the synthesis and physicochemical characterization of a new hybrid composite. Its main goals are evaluating the structure and studying the thermal and mechanical properties of the crosslinked polymeric materials based on varying chemical properties of the compounds. As an organic crosslinking monomer, bisphenol A glycerolate diacrylate (BPA.GDA) was used. Trimethoxyvinylsilane (TMVS) and N-vinyl-2-pyrrolidone (NVP) were used as comonomers and active diluents. The inorganic fraction was the silica in the form of nanoparticles (_NANO_SiO_2_). The hybrid composites were obtained by the bulk polymerization method using the UV initiator Irqacure 651 with a constant weight ratio of the tetrafunctional monomer BPA.GDA to TMVS or NVP (7:3 wt.%) and different wt.% of silica nanoparticles (0, 1, 3%). The proper course of polymerization was confirmed by the ATR/FTIR spectroscopy and SEM EDAX analysis. In the composites spectra the signals correspond to the C=O groups from NVP at 1672–1675 cm^−1^, and the vibrations of Si–O–C and Si–O–Si groups at 1053–1100 cm^−1^ from TMVS and _NANO_SiO_2_ are visible. Thermal stabilities of the obtained composites were studied by a differential scanning calorimetry DSC. Compared to NVP the samples with TMVS degraded in one stage (422.6–425.3 °C). The NVP-derived materials decomposed in three stages (three endothermic effects on the DSC curves). The addition of _NANO_SiO_2_ increases the temperature of composites maximum degradation insignificantly. Additionally, the Shore D hardness test was carried out with original metrological measurements of changes in diameter after indentation in relation to the type of material. The accuracy analysis of the obtained test results was based on a comparative analysis of graphical curves obtained from experimental tests. The values of the changes course of similarity in the examined factors, represented by those of characteristic coefficients were determined based on the Fréchet’s theory.

## 1. Introduction

Hybrid materials belong to the new generation of materials. They are defined as materials formed from a homogeneous mixture of inorganic and organic components, penetrating each other on a scale of less than 1 μm [1]. This structure makes it possible to design new products with great precision and to modify conventional materials at the nanometric or even molecular level. Most often organic-inorganic hybrid materials are obtained by mixing organic polymers (monomers) with inorganic compounds [2,3,4]. In this way, the obtained products are characterized by the properties that the individual components do not have [5,6,7,8].

The practically unlimited possibilities of combining organic and inorganic substances enable production of a wide range of new hybrid materials whose properties and applications will depend on the raw materials used in the synthesis [9,10].

Recently there has been a growing interest in organic-inorganic nanocomposites. The addition of relevant inorganic nanoparticles into the polymer matrix allows to obtain new high-performance materials with desired properties. To prepare such materials as a nanofiller, among others, non-metallic oxides (e.g., SiO_2_), metal oxides (e.g., TiO_2_, ZnO, Al_2_O_3_), metal particles (e.g., Au, Ag, Al, Fe) are used [11].

The synthesis of nanocomposites makes it possible to obtain materials with significantly improved mechanical (elastic modulus, strength, impact resistance), optical (optical transparency), electrical (conductivity), thermal (thermal resistance) properties. Moreover, they can reduce flammability and smoke release during combustion as well as limit the transmission of gases and solvents [12,13,14,15,16].

Composite materials are of interest specially in the aerospace, automobile, and energy industry due to their great strength and weight reduction compared to the conventional materials [17,18,19]. Most of the advanced polymer composites are derived from the epoxy resin based on bisphenol A. Epoxy composites are used for comprehensive adhesives, due to of their exceptional characteristics like good adhesion and, compatibility with a variety of materials. Although epoxy resins possess outstanding characteristics like good attachment to fiber reinforcements, better thermal, mechanical, and chemical properties, further research is being carried out to improve their e.g., resistance to damage, extreme environmental conditions, and high temperatures [20]. The processing of epoxy-based composites by the incorporation of microfillers (second phase) such as: graphite, silicon, rubber particles, carbide, alumina etc., was significantly improved. It was found that reducing the filler particles has a significant effect, due to composites properties improvement [17]. This phenomenon is especially visible when the nanoscale size of reinforcement (10^−9^ m) to the matrix from the epoxy resin was applied [21].

Parashar et al. described the synthesis and curing behavior of modified nanosilica particles and flammable properties of the epoxy resin-derived materials. The effect of nanosilica particles on the curing kinetics of epoxy resin was studied by means of DSC. The thermogravimetry analysis showed significant improvement in the thermal properties for the nanofiller samples [22]. Ghaemy et al. studied the effect of nanosilica on the kinetics of cure reaction and thermal degradation of epoxy resin based on diglycidylether of bisphenol-A (DGEBA), and 3,5-diamino-N-(4-(quinolin-8-yloxy)phenyl)benzamide (DQPB) as the curing agent. The study showed a slight catalytic effect of nanosilica particles on the curing reaction [23]. Pascual-Sanchez et al. described the effect of adding different amounts (0.5–3 wt.%) of nanosilica and organomodified montmorillonite to diglycidyl ether bisphenol A cured with isophorone diamine. The gel time measurements revealed that both fillers accelerated the curing reaction of DGEBA with IPDA. At room temperature, the addition of nanosilica increased both the stiffness (high storage modulus) and the toughness of composites. The improved properties can results from the interactions between the silanol moieties on the nanosilica surface and the polar groups in the epoxy resin chains [24]. In turn, the preparation of nanosilica filled dental composites derived from 51 wt.% bisphenol-A glycidyl methacrylate and 46.5 wt.% triethylene glycol dimethacrylate was reported in the paper by Sonal et al. [25]. The composites were modified by varying a filler content from 0 to 9 wt.% The results indicated that the wear resistance and hardness were improved with the increase in the filler. Similar observations are presented in the paper by Kumar et al. [26].

Due to the fact that the studies related to the application of nanosilica as a filler are mainly carried out in the systems based on epoxy resins derived from diglycidyl ether bisphenol A, the attempt was made to apply this filler in the acrylate monomers. In this paper the bisphenol A glycerolate diacrylate (BPA.GDA) was synthesized with trimethoxyvinylsilane (TMVS) or N-vinyl-2-pyrrolidone (NVP) hybrid composites, by the bulk polymerization method, with the addition of silica nano powder as a nanofiller. The BPA.GDA monomer has aromatic rings and two pairs of acrylate groups capable of crosslinking while TEVS (or NVP) is used as active solvents and co-monomers. The UV-initiator (2,2-dimethoxy-2-phenylacetophenone) was used in the free radical polymerization reaction. The physico-chemical characterization especially that of spectroscopic, thermal, and mechanical properties of the proposed organic-inorganic hybrid composites is discussed in detail.

## 2. Materials and Methods

### 2.1. Materials and Chemicals

Bisphenol A glycerolate (1 glycerol/phenol) diacrylate (BPA.GDA) (molecular mass: 484.54 g·mol^−1^; viscosity: 2000–4000 cP (65 °C); density at 25 °C: 1.18 g/cm^3^; refractive index: 1.557, trimetoxyvinylsilane (TMVS) (molecular mass: 148.23 g·mol^−1^ density 20 °C: 0.970 g/cm^3^; refractive index: 1.390), N-vinyl-2-pyrrolidone (NVP) (molecular mass: 111.14 g·mol^−1^; density 20 °C: 1.04 g/cm^3^; refractive index: 1.512, silica (nonpower, specific surface area BET = 175–225 m^2^/g) and 2,2-dimethoxy-2-phenylacetophenone (Irgacure 651, IQ) were from Sigma-Aldrich, Germany.

### 2.2. Synthesis of Composites

For the synthesis of copolymers, the free radical, bulk polymerization method was used. Due to the large viscosity of BPA.GDA monomer, it is necessary to add active diluents in order to obtain the required processing parameters of the liquid composition. First, the appropriate amount of BPA.GDA, and one of the active solvents (TMVS or NVP) in the weight proportions 7:3 were added into the glass vessel, slowly mixed and transferred to the heating chamber to deaerate the samples (60 °C). Next, a suitable amount of nano filler (1 or 3 wt.%) SiO_2_ in the form of nano powder was transferred to the mixture and slowly stirred to make sure that the added filler was homogeneously distributed throughout the sample. Finally, the UV initiator was added to the liquid composition (1 or 1.5 wt.%). The whole mixture was placed back in the heating chamber at 60 °C until the sample was completely deaerated. The liquid compositions were poured into 100 × 80 × 2 mm special molds composed of two glass panes and a Teflon spacer protected with non-stick grease [27].

**Figure 1:**
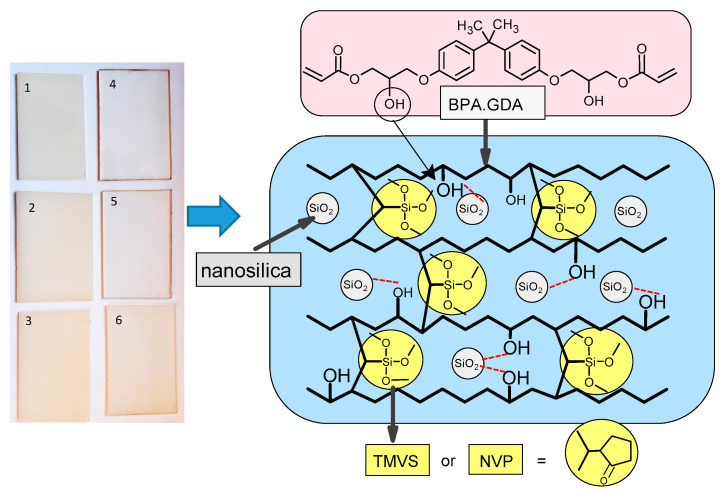
Chemical structure of monomers and the proposed fragment of the composite structure.

The compositions were placed inside the irradiation chamber where they were exposed (30 min) to UV light with four mercury lamps each of 40 W at room temperature. The experimental parameters of the syntheses are presented in Table 1. The proposed fragment of the composite structure is presented in Figure 1.

### 2.3. Characterization Methods

The attenuated total reflection (ATR) was recorded using the infrared Fourier transform spectroscopy using a TENSOR 27, with the Bruker spectrometer, equipped with a diamond crystal (Ettlingen, Germany). The spectra were recorded in the range of 600–4000 cm^−1^ with 32 scans per spectrum at a resolution of 4 cm^−1^.

Microanalysis of elements was carried out by using scanning electron microscope EDAX ZAF Quantification, Standardless, (FEI Company, Hillsboro, FL, USA), equipped with detector Type: Octane Elect Plus, Resolution: 126.10, Lsec: 337.

Differential scanning calorimetry (DSC) curves were obtained with the use of a DSC Netzsch 204 calorimeter Netzsch (Günzbung, Germany). The measurements were made in the aluminum pans with a pierced lid and the sample mass was approx. 10 mg in the nitrogen atmosphere (30 cm^3^/min). Dynamic scans were performed at a heating rate of 10 °C/min in the temperature range −30–510 °C. An empty aluminum crucible was used as the reference.

The fragments of the solid composites were studied using the optical microscope Morphologi G3 (Malvern, UK).

The hardness of the materials was measured by the Shore method using the electronic hardness testing apparatus model: Art. 13 Affri (Induno Olona, Italy), with the D scale head at 23 °C. All hardness test results were read after 15 s. The NIKON Eclipse LV100ND microscope (Tokyo, Japan) was used to observe the depressions created by the hardness test. Metrological measurements: the diameter of the resulting indentation after the needle removal and the height of the resulting border were performed using the NIKON NIS-Elements software (Tokyo, Japan). The samples in the form of specimens cut from the pressed sheet form (10 mm × 60 mm × 2 mm) were subjected to the strength tests during the uniaxial tensile strength. The tests were carried out according to EN-ISO 527, the test speed was 50 mm/min at 23 °C. In order to examine the bending strength, a test including the three-point bending tests (EN-ISO 170, ASTM D-790 standards) was carried out. All tests were performed using the Zwick/Roell Z010 universal tensile-testing machine (Ulm, Germany).

## 3. Results

In the synthesis of polymeric nanocomposites, the crosslinking monomer BPA.GDA possessing two vinyl groups was used. In this way a rigid, chemically resistant polymer network was formed. Active diluents in the form of TMVS and NVP were used to provide the appropriate consistency (viscosity) of the polymerization mixture. Nano powdered SiO_2_ was applied as a nanofiller. Additionally, BPA.GDA including hydroxyl groups in its structure could affect with the silica nanoparticles by hydrogen bonds and weaker intermolecular interactions, contributing to its stronger incorporation into the polymeric network. Similar interactions are possible with active solvents as these compounds also possess ether and carbonyl atoms of oxygen in their structure. The above mentioned hydroxyl bonds are marked in Figure 1 (red line). The proposed system of monomers and nanofiller in the form of SiO_2_ results in obtaining hybrid nanocomposites having inorganic fragments composed of silica nanoparticles and silane groups (TMVS) as well as organic part from acrylic derivative of bisphenol A and NVP.

### 3.1. ATR/FTIR Analysis

ATR/FTIR spectroscopy was applied to prove the proper course of copolymerization reaction. The formation of a copolymer network is associated with the contents of both monomers, BPA.GDA and NVP (or TMVS). In order to confirm the proper course of polymerization as well as the presence of nanosilica in the composite structure, the spectra of starting NVP and TMVS monomers and _NANO_SiO_2_ are shown in Figure S1 (Supplementary Material). In the NVP spectra the vibrations originating from the stretching C=C vibrations at 1625 cm^−1^, C-H in-plane deformation at 1487 cm^−1^, C-H out-of-plane deformation vibrations at 981 and 843 cm^−1^ vinylene group are noticeable. In the TMVS spectra a signal from the C-H in-plane deformation vibrations of the vinylene groups at 1410 cm^−1^ appeared. Additionally, a strong signal at 767 cm^−1^ coming from the C-H vibrations of vinylene group is visible. After polymerization these signals disappear. The characteristic vibrations coming from the copolymerized compound are observed in Figures S2 and S3 (Supplementary Material). Additionally, the most important signals are marked. In the spectrum of NVP-derived copolymers the strong C=O absorption peak from the amide group at 1672–1675 cm^−1^ is clearly visible. A medium strong absorption band of the C-N stretching vibrations appears at 1246 cm^−1^, and the C-H sym. deformation vibrations from amides are observed near 1422 cm^−1^ [28]. In the case of TMVS derivatives in the spectrum the characteristic bands at 1053–1100 cm^−1^ associated with the asymmetrical stretching vibrations of Si–O–C groups and Si–O–Si stretching ones are visible which confirms the incorporation of TMVS into the structure of polymerized composite [29]. The existence of stretching vibrations from the C–H of methyl (-CH_3_) ~2960 cm^−1^, ethyl (-CH_2_–CH_3_) ~2900 cm^−1^, carbonyl (C=O) ~1730 cm^−1^, and hydroxyl (-OH) ~3380 cm^−1^ groups derived from the BPA.GDA molecule is also observed for all spectra. The stretches of aromatic ring are visible at ~1600, 1500, and 1450 cm^−1^, respectively [30].

### 3.2. Microanalysis Study

**Figure 2:**
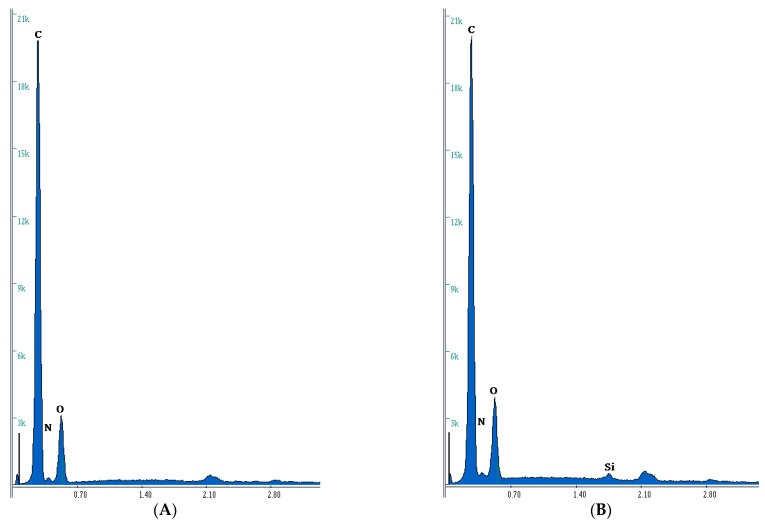

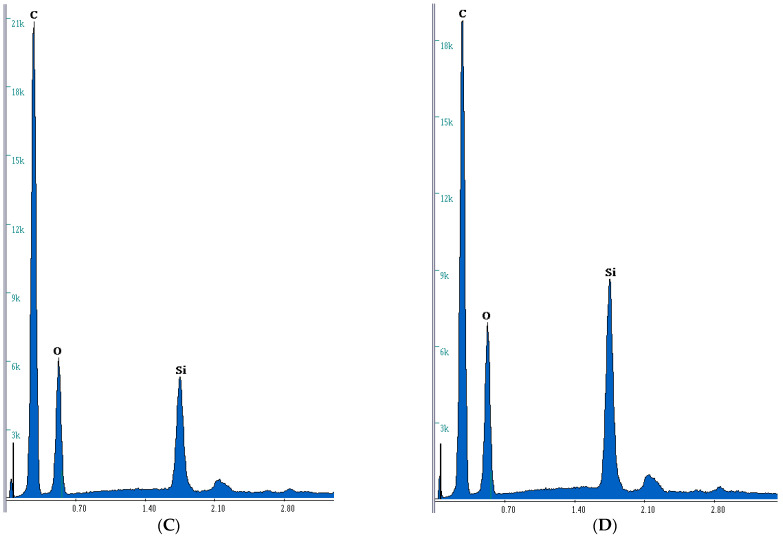
Microanalysis report: (**A**): BPA.GDA-NVP0%; (**B**): BPA.GDA-NVP3%; (**C**): BPA.GDA-TMVS0%; (**D**): BPA.GDA-TMVS3%.

In order to confirm the chemical structure of composites and the presence of selected atoms (especially Si), elementary analysis was performed using the scanning electron microscope (EDAX SEM). Table 2 shows the microanalysis data with weight (wt.) and atomic (at.), percentage quantity of atoms presented in structure of composites: C, Si, O, and N, for 0 and 3 wt.% of nanofiller. In Figure 2A, microanalysis report in graphical form is also presented. This research confirms the presence of Si atoms on the surface of the materials. The highest values were obtained for the material with the TMVS and 3% addition of nanosilica (6.38 wt.%). This value is obviously due to the presence of silicon atoms in the monomer structure. But after adding the nanosilica to the composites, the silicon content increases from 4.27 to 6.38% wt. from TMVS-derived materials. Additionally, in Figure S7 (Supplementary Material) the SEM photos of the material are illustrated. After addition of nanofiller some changes in the photos are visible (bright dots). These are most likely agglomerates of nanosilica. The Si content in NVP-derived materials may be undervalued because the measurement is only on the surface of the composites [29].

### 3.3. DSC Analysis

**Figure 3:**
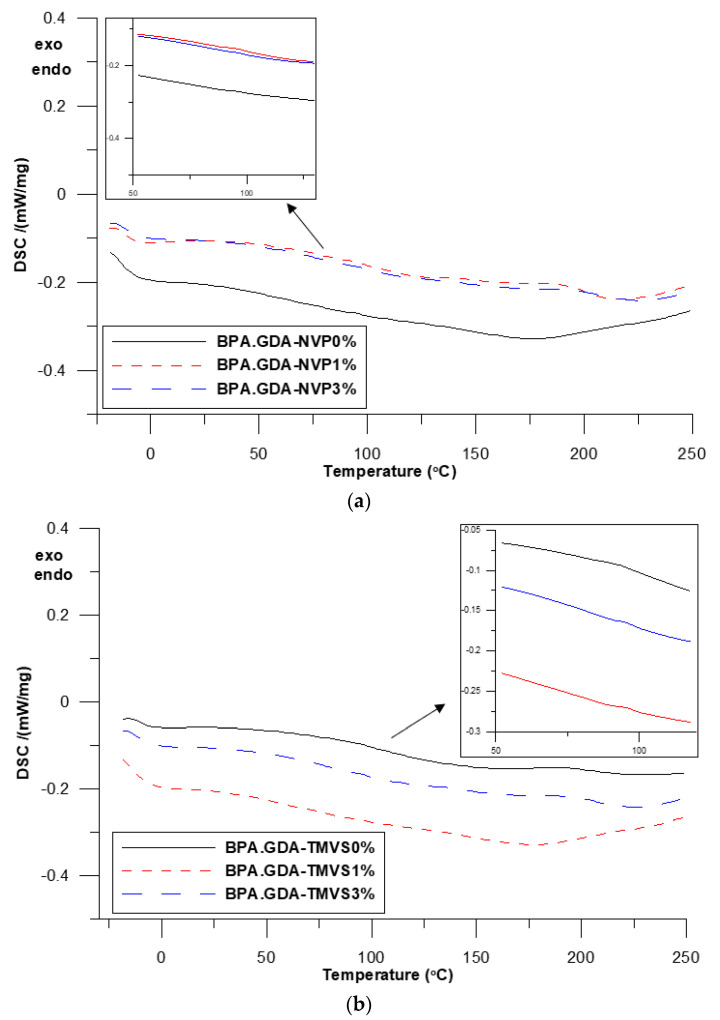
DSC curves in the temperature range: −30 to 250 °C, NVP-derived composites (**a**) and TMVS-derived composites (**b**).

**Figure 4:**
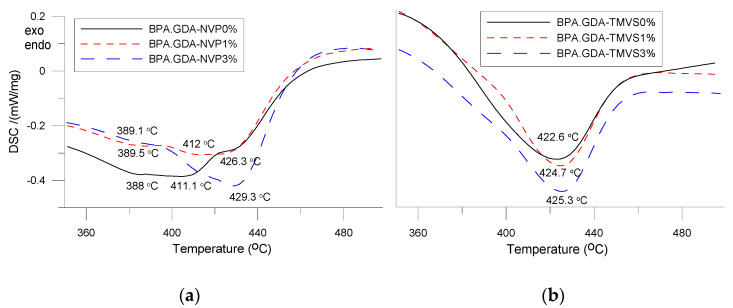
DSC curves in the temperature range: 350 to 500 °C, NVP-derived composites (**a**) and TMVS-derived composites (**b**).

DSC measurements were made in the course of one heating cycle at the temperatures ranging from −30 to 510 °C. The numerical data of the DSC analysis is presented in Table 3. The DSC curves of the obtained hybrid materials are shown in Figure 3a,b and Figure 4.

In Figure 3a,b the curves for the samples in the range −30 to 250 are visible, additionally the region concerning the glass transition temperature was expanded. However, the vitreous transition is broad and there is no clear inflection in the curves. As one can see, no significant changes in the samples behavior are observed in this temperature region. Only at about 150–160 °C a small endothermic effect associated with the evaporation of unreacted monomer is visible. The samples are characterized by a good degree of double bond conversion which is evidenced by the lack of a pronounced exothermic effect (around 200 °C) from the double bond crosslinking. In Figure 4a,b the curves of the samples in the range 350–500 °C are presented. Some differences related to the course of curves for the NVP and TMVS-based materials can be seen. For the BPA.GDA-TMVS-derived materials one endothermic effect with the maximum at 422.6–425.3 °C was observed. This endothermic effect resulted from the total thermal degradation of the samples [31,32].

The addition of silica nanofiller increases the temperature of composites maximum degradation insignificantly. The DSC curves of the composites with NVP have a different course, and degradation occurs in several stages. The reason for this phenomenon can be the fact that during polymerization, NVP homopolymer fragments are formed. These parts decompose earlier due to the non-aromatic nature of the N-vinyl-2-pyrrolidone structure. The maxima of the endothermic effects peaks occur in the temperature range from 388 to 429 °C. The addition of nano filler affects the thermal resistance of the obtained materials positively [27].

### 3.4. Mechanical Properties

#### 3.4.1. Hardness Tests

**Figure 5:**
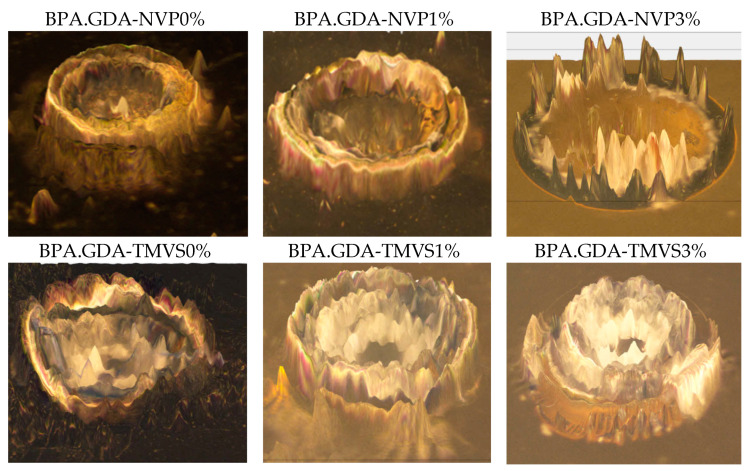
Photographs of depressions obtained during the hardness measurements made by means of the Nikon microscope.

The standard hardness test was carried out using the Shore D method (Table 4). The test consisted in determining the resistance of the material to pressing with a defined force into the surface of the sample indenter in the form of a needle with a cross-sectional area of 1 mm^2^. In order to provide a more comprehensive description of the phenomena related to hardness, i.e., the resistance of the material to penetration into it by the indenter of a hardness tester there were made additional measurements, developed as the author’s method. The additional testing procedure with the use of an optical metrology package consisted in the assessment of the effects of inserting an indenter needle into the material surface. Immediately after the hardness measurement, the sample was placed under the objective of a microscope operating in the reflected light. The shape and dimensions of the indentation obtained after removing the steel needle from the sample were measured (Figure S4, Supplementary Material). In this way, in addition to the numerical value of hardness, there were obtained images showing the unique characteristics of the studied polymeric composition, from which the individual samples were prepared. For better visualization of the hardness research the photos of depressions obtained during the hardness measurements are presented (Figure 5).

The hardness of the tested samples varied in the range 82.17–92.18 °Sh. The addition of the nanofiller into the BPA.GDA-NVP compositions caused an increase in hardness by about 9%, but this change is similar in both concentrations (1 and 3%). The lack of increase in hardness with the changing amount of nanofiller is most likely related to the presence of the aerated nanofiller, characterized by a relatively small mass at a large volume.

For the TMVS-derived composites, the hardness is slightly smaller than expected (increase by c.a. 4%). The presence of Si atoms in the TMVS structure does not increase the hardness of the material compared to the NVP-derivatives. This is probably due to the spatial, developed structure of TMVS compounds which results in a less compact construction of the composite. Additionally, in Figure S5 (Supplementary Material) the metrological measurements of the diameter and height of a depression in the samples after removal of a hardness tester needle are presented.

The photos presented in Figure 5 show the differences in the behavior of a material under an external force. The magnitude of the depression and its shape pointed out how the surface of a sample would behave after breaking the cohesion in the surface layer of the material, i.e., indirectly about larger viscosity of the composition. The addition of the 1 to 3% nanofiller increases the cohesion of the material and therefore, improves resistance to surface damage caused by the concentrated force.

#### 3.4.2. Mechanical Strength Tests during Uniaxial Tensile

Uniaxial tensile testing is the basic method for finding the durability characteristics such as Young’s modulus, tensile strength, plasticity limit, extension at break, etc., of construction materials. Testing was based on the uniaxial tensile of a sample attached to holders, one of which moves at a constant speed in a vertically upwards direction (Figure S5, Supplementary Material). The measurements are made at a specified temperature of 23 °C until the sample breaks. During the test the dependence of changes in the tensile force on the sample deformation is monitored.

**Figure 6:**
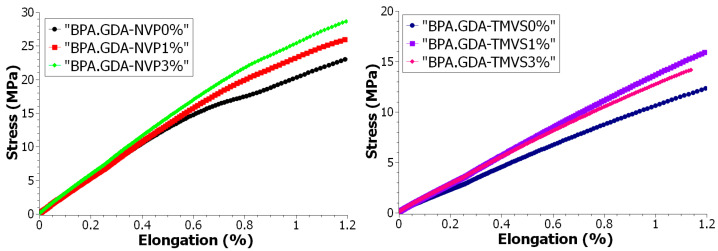
Graph of stress value as a function of elongation in the range of up to 1.2% of relative materials.

**Figure 7:**
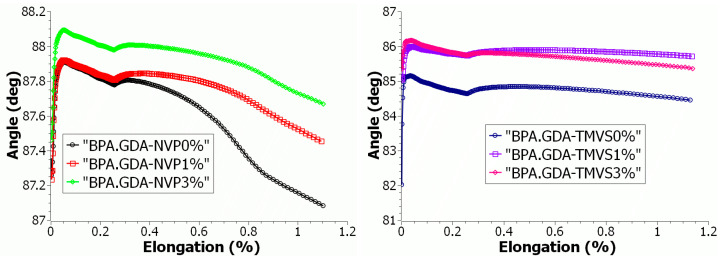
Graph of angle transformation as a function of relative materials elongation.

The results of the tests are presented in Table 5, while the graphs (Figure 6 and Figure 7) show the progression of the changes of strain values in the function of deformation in the form of curves obtained from the uniaxial tensile test and the directional factor of the straight line tg alpha. Modification with the nanofiller of both BPA.GDA-NVP and BPA.GDA-TMVS caused an increase of modulus by about 10% and 26%, respectively at a higher ratio. The short-term endurance on the strength of BPA.GDA-NVP increased by slightly more than 16% with the introduction of the nanofiller 1% sample B. The addition of the nanofiller to BPA.GDA-TMVS resulted in a decrease of short-term strength to tensile by 6.2% in the smaller range of its content in the material and by nearly 46% in the case of its 3% content. In the case of both materials with an increase of the nanofiller amount, relative extension was decreased at the yield point by over 25% in BPA.GDA-NVP and by as much as 70% in the case of BPA.GDA-TMVS containing 3% of the nanofiller. The introduction of nanofiller also resulted in an increase of the intensity of the increasing strain in the function of extension in the range to 1.2% of relative deformation (Figure 5).

#### 3.4.3. Mechanical Durability Tests during Bending

The other method used for determination of the mechanical characteristics of the samples was the bending test. The most typical example of the action of external bending forcing is the three-point bending. The bending strength represents the greatest strain created in the sample material at the moment of damage—breaking. Owing to this it is possible to determine the values of the elastic modulus at bending, conventional limit of elasticity, strength at bending, and deformation upon bending. Table 6 presents the results of the mechanical properties determination during bending.

In the following diagram the progress and characteristics of the curves obtained from the measurement points are compared as regards changes in the value of strain during stretching in the strain function during bending. The functional dependencies allow for a more accurate assessment of the features and statistical as well as comparative analyses of the behavior of the material from which the individual samples are made as a result of value changes and type of external forces and pressures. In accordance with the expectations, the addition of the nanofiller resulted in an increase of the flexural modulus at bending, strain in the elastic range, and σ_fM_. In the case of C, BPA.GDA-NVP samples at the 3% nanofiller content, more than double (137.5%) increase of modulus, more than 88% increase of strain in elastic range, and 21% increase of σ_fM_ value were gained. Similarly, the introduction of 3% nanofiller to the BPA.GDA-TMVS sample resulted in an increase of modulus by nearly 58%, strain in the elastic range by 43% while σ_fM_ decreased by just over 15%.

**Figure 8:**
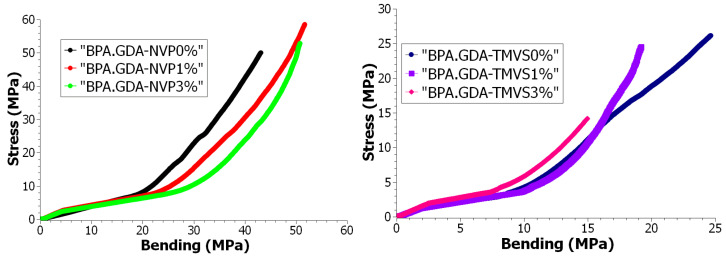
Graph of stress value as a function of elongation in the range of up to 1.2% and stress related to the bending test results for the TMVS materials.

There were made the dependence diagrams for the change of strain in the deformation function for each individual measurement. Based on the obtained results, there were elaborated the graphical dependences of the value of strain during the uniaxial tensile in the strain function during bending. The results were confined to the area of Hook’s law action, i.e., in the range of deformations at the plastic limit of the analyzed materials. The graphical dependences are shown in Figure 8.

Due to the fact that the graphical dependencies show the characteristics and the course of the material behavior during the action of the external forcing indirectly, it was assumed that the analysis and the comparison of the physical sizes were based on the evaluation of the similarity of the position of the characteristic points on the obtained dependencies.

The results of selected mechanical properties obtained with the use of a testing machine allowed for the assessment of usable features and characteristics of the tested materials. The results are presented in Table 7 and Figure 7 and Figure 8. The dependence plots were made for the change of stress value as a function of strain for each single measurement. There were determined the graphical relations of the value of stress throughout the uniaxial tension as a function of stress during bending. The results were confirmed to the area of Hook’s law action, i.e., in the range of deformations in the yield stress of the tested materials. Graphical relationships are presented in the graphs. The graphical relations show the characteristics and the course of the material behavior during the action of the external forcing indirectly. Therefore, it was assumed that the analysis and comparison of the physical quantities can be based on the evaluation of the similarity of the distribution of the characteristic points on the obtained curves.

For this purpose, there was applied the characteristic index known in the literature as the Fréchet distance [33]. The Fréchet distance is a measure of the similarity between the curves taking into account the position and order of points on the curves.

Let us define, according to [34], a curve as a continuous mapping *f*:[a, b] → V, where a, b ∈ R and a ≤ b and (V, *d*) is a metric space. For two curves defined as *f*:[a, b] → V and *h*:[a’, b’] → V the Fréchet distance is defined as:

$$\delta_{F}\left(f,h\right)=\underset{}{\underset{\alpha ,\beta \;t\in \left[0,1\right]}{\inf}\;\max\;d\left(f\left(\alpha (t)\right),\;h\left(\beta (t)\right)\right)}$$

where *α* (respectively *β*) is an arbitrary continuous nondecreasing function from [0, 1] onto [a, b] (respectively [a’, b’]).

The Fréchet distances for stress tests calculated based on the measurement results are given in Table 7 and Table 8. It can be noticed that the values (distance) were observed for the NVP and TMVS family and are presented in Figure 8.

## 4. Conclusions

The synthesis of hybrid composites using the bulk polymerization method and UV-initiator was discussed. As a monomer, bisphenol A glycerolate diacrylate (BPA.GDA) and trimethoxyvinylsilane (TMVS) or N-vinyl-2-pyrrolidone (NVP) with different chemical structures was applied. The compositions were successfully modified by the addition of nanosilica from 0 to 3 wt.% As a result of the polymerization reactions there were obtained crosslinked and rigid composites. As indicated by SEM EDAX analysis, after adding the nanosilica to the composites, the silicon content increases from 4.27 to 6.38 wt.% for the TMVS derived materials. The maxima of the endothermic effects peaks occur in the temperature range from 388 to 429 °C. The composites with the addition of TMVS decompose in a single step. The addition of nanosilica to the NVP-based composites increases the system homogeneity.

The obtained materials hardness was in the range of 82.17–92.18 °Sh. The addition of the nanofiller into the composites caused an increase in hardness (4–9%). The chemical structure of monomers as well as nanofiller had a great effect on this parameter. The presence of silica atoms in the trimetoxyvinylsilane structure does not increase the hardness of the material (compared to the NVP-derivatives). It can be caused by the spatial structure of TMVS monomer which results in a less compact construction of the composite. Additionally, there were taken photos of the examined areas of the composites using the optical microscopy. With the addition of nanosilica the cohesion forces increase which can be seen in the pictures as non-uniform borders.

Characteristic features and comparison of mechanical properties are compared in order to select the best combination of materials based on the results of the tensile strength and bending tests. This assessment can be made traditionally comparing the values of individual characteristic quantities. However, in these comparisons mainly average values are taken from several measurements. More information about the behavior of a specific material under the influence of the external force in this case throughout the action of the external force can be obtained by the direct analysis from the graphic dependencies using the Fréchet method. This gives a more complete description of the material properties of silica nanoparticles and causes the analysis of the test accuracy to take place during the entire process of material strain deformation.

The Fréchet distance analytical analysis shows the smallest values (shortest distance) on the NVP (1% or 3%) and TMVS (1% or 3%) family materials curves. That indicates that adding the NANO filler causes similar behavior of the studied composites.

## Figures and Tables

**Table 1:** Experimental parameters of the synthesis.

No.	Composite	BPA.GDA	NVP	TMVS	_NANO_SiO_2_	IQ
		g			wt.%	
1	BPA.GDA-NVP0%	8.4	3.6	0	0	1
2	BPA.GDA-NVP1%	8.4	3.6	0	1	1
3	BPA.GDA-NVP3%	8.4	3.6	0	3	1.5
4	BPA.GDA-TMVS0%	8.4	0	3.6	0	1
5	BPA.GDA-TMVS1%	8.4	0	3.6	1	1
6	BPA.GDA-TMVS3%	8.4	0	3.6	3	1.5

where: TMVS– trimetoxyvinylsilane; NVP– N-vinyl-2-pyrrolidone; _NANO_SiO_2_– silica nanoparticles; IQ– Irgacure 651.

**Table 2:** Microanalysis data.

No.	Composite	C	Si	O	N
		wt.%/at.%	wt.%/at.%	wt.%/at.%	wt.%/at.%
1	BPA.GDA-NVP0%	72.93/77.82	-/-	22.70/18.19	4.37/3.99
3	BPA.GDA-NVP3%	69.13/74.43	0.37/0.17	23.81/19.25	6.64/6.13
4	BPA.GDA-TMVS0%	69.88/76.73	4.27/2.01	25.76/21.23	-/-
6	BPA.GDA-TMVS3%	66.87/74.59	6.38/3.04	26.67/22.33	-/-

**Table 3:** Differential scanning calorimetry (DSC) data of the obtained materials.

Sample	Composite	T_g_	T_max1_ (°C)	T_max2_	T_max3_
A	BPA.GDA-NVP0%	89.1	388	411.1	426.0
B	BPA.GDA-NVP1%	91.8	389	-	426.3
C	BPA.GDA-NVP3%	98.0	-	-	429.3
D	BPA.GDA-TMVS0%	99.1	-	-	422.6
E	BPA.GDA-TMVS1%	100.6	-	-	424.7
F	BPA.GDA-TMVS3%	107.1	-	-	425.3

**Table 4:** Hardness values.

Sample	Composites	Mean Value, Shore Degrees, D Scale	Diameter µm	High µm
A	BPA.GDA-NVP0%	84.28	262.80	60
B	BPA.GDA-NVP1%	92.12	283.12	74
C	BPA.GDA-NVP3%	92.18	299.34	78
D	BPA.GDA-TMVS0%	82.17	489.12	60
E	BPA.GDA-TMVS1%	82.28	585.74	73
F	BPA.GDA-TMVS3%	85.65	596.56	75

**Table 5:** Results of strength tests during the uniaxial tensile.

Samples	Composites	Young’s Module E_t_	Tensile Strength σ_m_	Elongation ε_m_	Sample Width b	Sample Thickness h
		MPa	MPa	%	mm	mm
A	BPA.GDA-NVP0%	2542.80	50.12	3.2	10.62	2.12
B	BPA.GDA-NVP1%	2576.15	58.53	3.5	10.09	2.06
C	BPA.GDA-NVP3%	2794.53	52.85	2.6	9.15	2.01
D	BPA.GDA-TMVS0%	1038.56	26.11	3.8	8.00	2.92
E	BPA.GDA-TMVS1%	1322.04	24.50	2.2	8.85	1.97
F	BPA.GDA-TMVS3%	1309.18	14.16	1.1	9.56	2.05

**Table 6:** Mechanical properties bending test results.

Samples	Composites	Young’s Module E_f_ MPa	Bending Strength σ_fM_ MPa	Elongation ε_fM_ %	Sample Thickness h mm	Sample Width b mm	Cross Section Area A_0_ mm^2^
A	BPA.GDA-NVP0%	1770.02	103.96	2.9	2.09	10.07	21.04
B	BPA.GDA-NVP1%	3787.36	110.01	3.3	2.08	8.85	18.40
C	BPA.GDA-NVP3%	4204.99	125.83	3.6	2.00	9.91	19.82
D	BPA.GDA-TMVS0%	1514.66	42.51	3.2	2.92	10.06	29.37
E	BPA.GDA-TMVS1%	1725.73	45.27	3.2	2.01	9.48	19.05
F	BPA.GDA-TMVS3%	2389.02	35.84	1.4	2.06	9.86	20.31

**Table 7:** Stress Fréchet distance curves for the two material families.

Compared Curves	Fréchet Distance
NVP family	
BPA.GDA-NVP0%/BPA.GDA-NVP1%	2.959537
BPA.GDA-NVP0%/BPA.GDA-NVP3%	5.536141
BPA.GDA-NVP1%/BPA.GDA-NVP3%	2.576815
TMVS family	
BPA.GDA-TMVS0%/BPA.GDA-TMVS1%	3.524791
BPA.GDA-TMVS0%/BPA.GDA-TMVS3%	2.39698
BPA.GDA-TMVS1%/BPA.GDA-TMVS3%	1.128088

**Table 8:** Bending Fréchet distance curves for two material families.

Compared Curves	Fréchet Distance
NVP family	
BPA.GDA-NVP0%/BPA.GDA-NVP1%	9.13941
BPA.GDA-NVP0%/BPA.GDA-NVP3%	8.486294
BPA.GDA-NVP1%/BPA.GDA-NVP3%	0.7185488
TMVS family	
BPA.GDA-TMVS0%/BPA.GDA-TMVS1%	5.132055
BPA.GDA-TMVS0%/BPA.GDA-TMVS3%	9.985838
BPA.GDA-TMVS1%/BPA.GDA-TMVS3%	5.002452

## Data Availability

Any data available.

## Supplementary Materials

The following are available online at https://www.mdpi.com/article/10.3390/ma14237431/s1, Figure S1: ATR-FTIR spectra for active solvents and nanosilica, Figure S2: ATR/FTIR spectra for the NVP copolymers, Figure S4: View of the measuring head during the hardness test, Figure S5: Example of metrological measurement of diameter and height of a depression in the samples after removal of a hardness tester needle, Figure S6: Appearance of the attached sample in the holder of the endurance machine after the test, Figure S7: SEM photos of the studied materials: A: BPA.GDA-NVP0%; B: BPA.GDA-NVP3%; C: BPA.GDA-TMVS0%; D: BPA.GDA-TMVS3%.
