# Supplementary material for: The Influence of Silica Nanoparticles on the Thermal and Mechanical Properties of Crosslinked Hybrid Composites
Source: Materials (Basel). 2021 Dec 3;14(23):7431. doi: 10.3390/ma14237431 (PMC8659203; doi:10.3390/ma14237431)
Supplement: Supplementary file 1 [file materials-14-07431-s001.zip › materials-1457883-supplementary.pdf]

Supplementary Information

The influence of silica nanoparticles on the thermal and mechanical properties of crosslinked hybrid composites

Tomasz Klepka[1*], Beata Podkościelna[2], Dariusz Czerwinski[3] and Bronisław Samujło[1]

[1] Lublin University of Technology, Faculty of Mechanical Engineering, Department of Technology and Polymer Processing, Nadbystrzycka 36, 20-618 Lublin, Poland; t.klepka@pollub.pl
[2] Maria Curie-Skłodowska University, Faculty of Chemistry, Institute of Chemical Science, Department of Polymer Chemistry, M. Curie-Skłodowska Sq. 3, 20-031 Lublin, Poland; beata.podkoscielna@mail.umcs.pl
[3] Lublin University of Technology, Department of Computer Science, Nadbystrzycka 36, 20-618 Lublin, Poland; d.czerwinski@pollub.pl
* Correspondence: t.klepka@pollub.pl

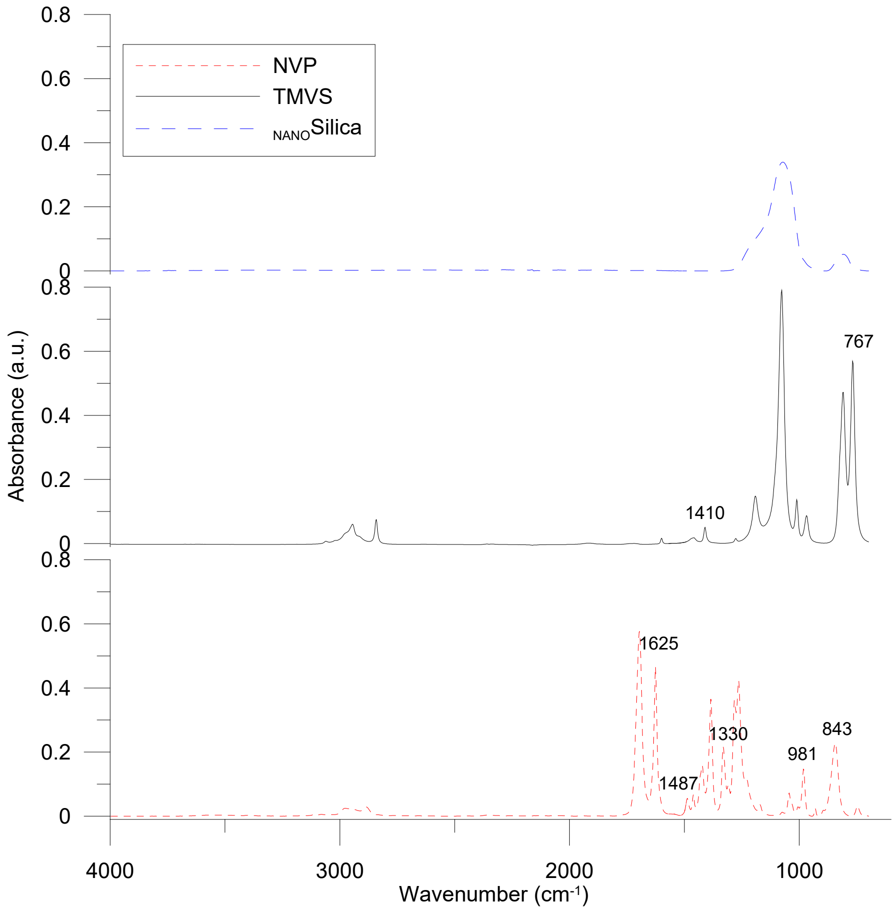

Figure S1. ATR-FTIR spectra for active solvents and nanosilica.

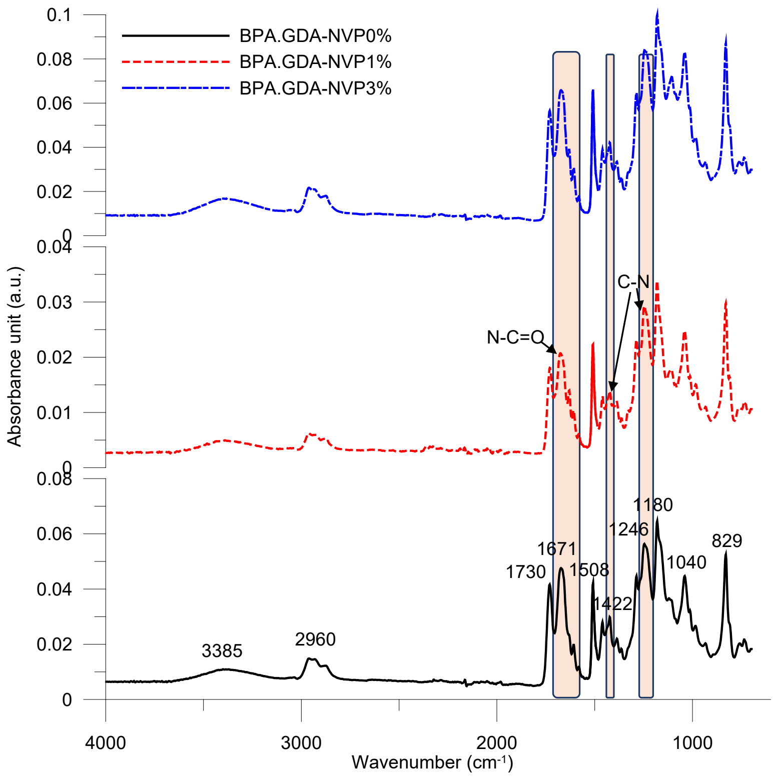

Figure S2. ATR/FTIR spectra for the NVP copolymers.

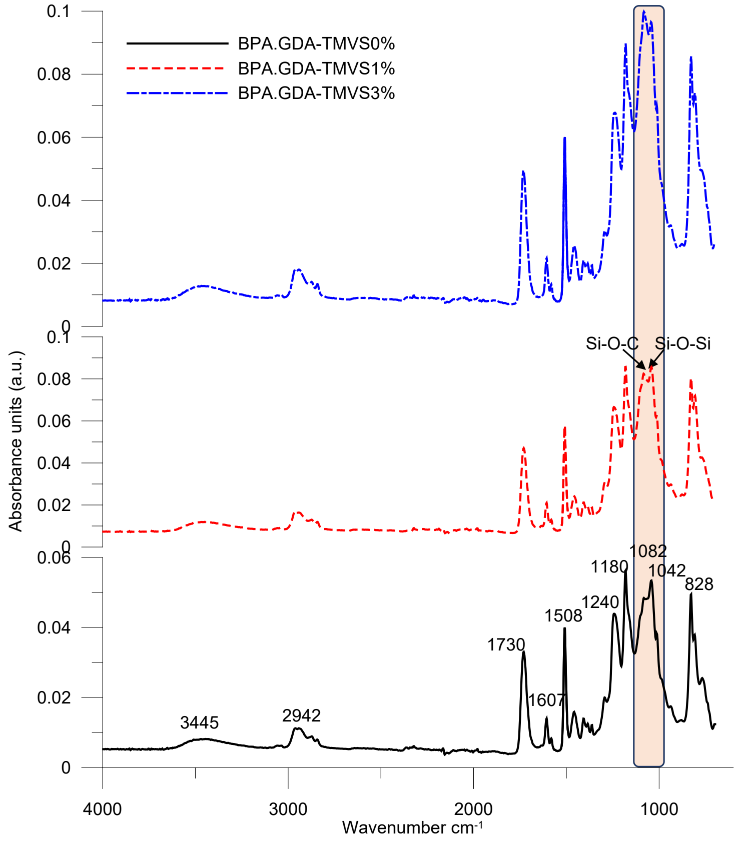

Figure S3. ATR/FTIR spectra for the TMVS copolymers.

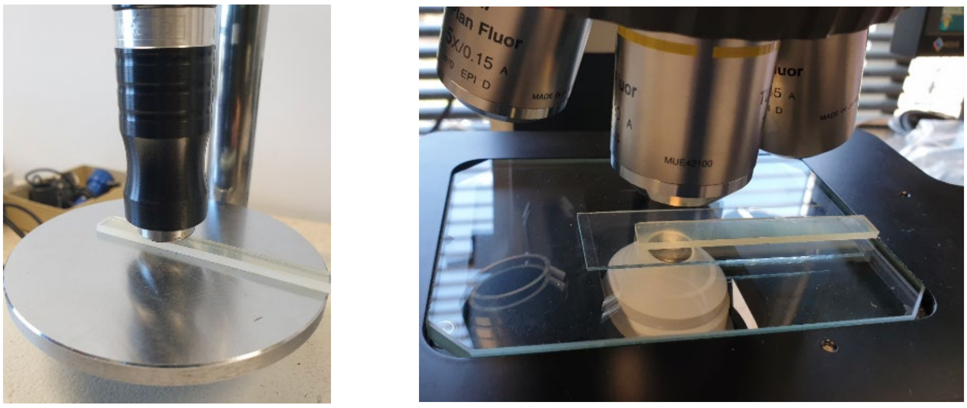

Figure S4. View of the measuring head during the hardness test.

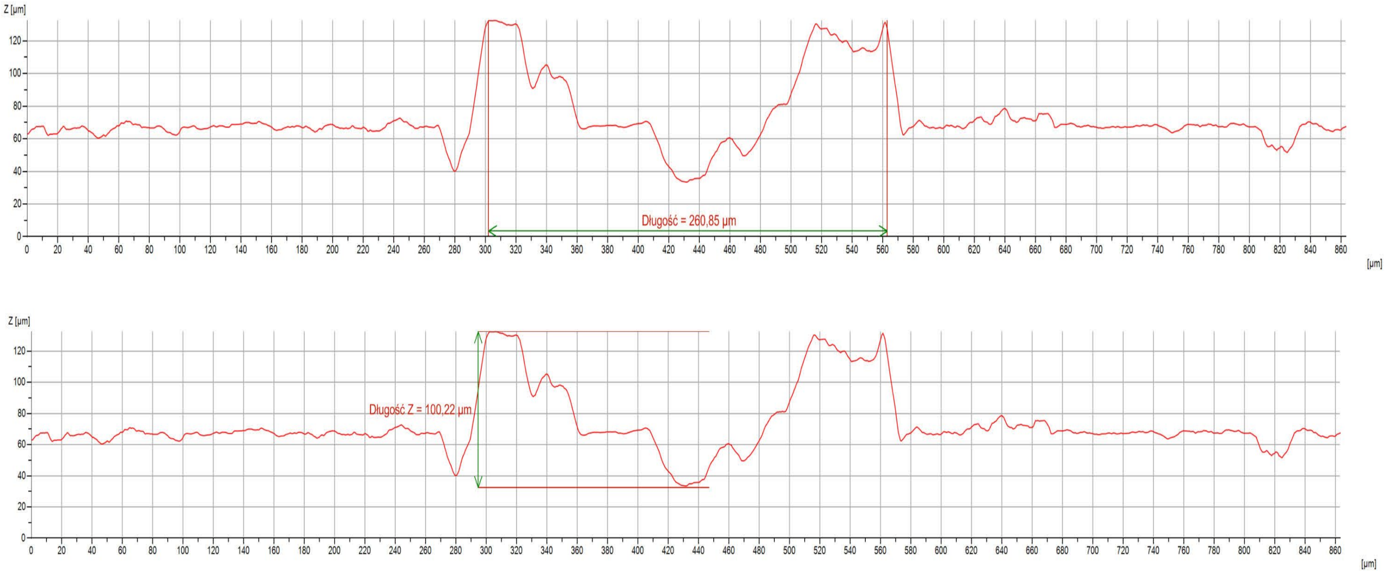

Figure S5. Example of metrological measurement of diameter and height of a depression in the samples after removal of a hardness tester needle.

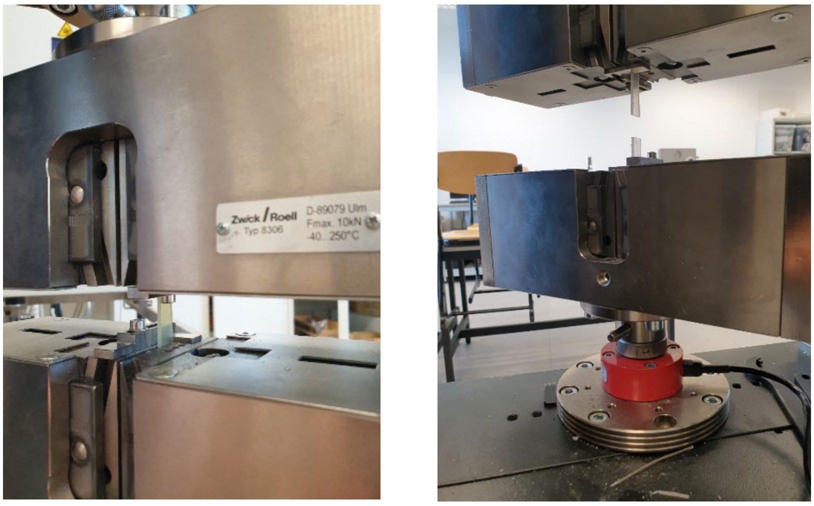

Figure S6. Appearance of the attached sample in the holder of the endurance machine after the test.

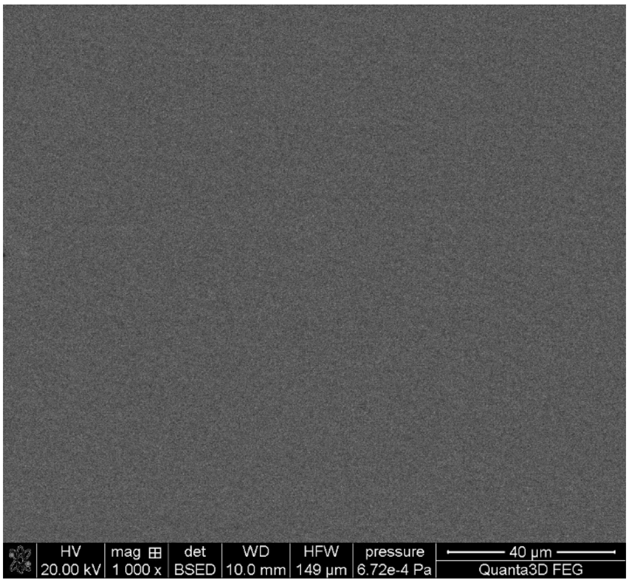

A

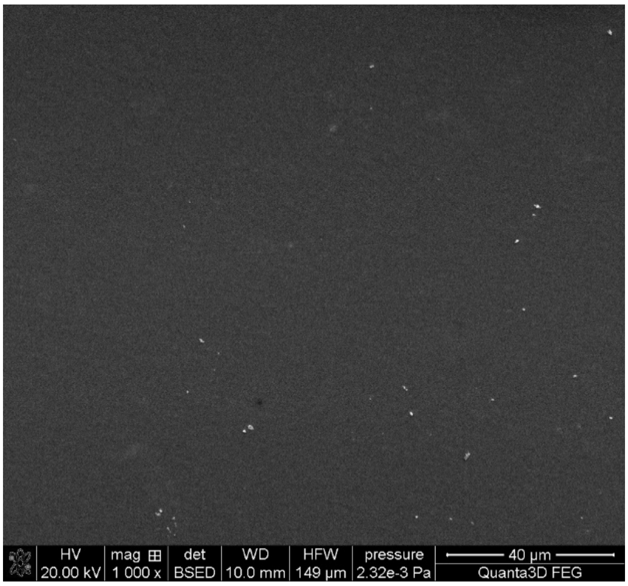

B

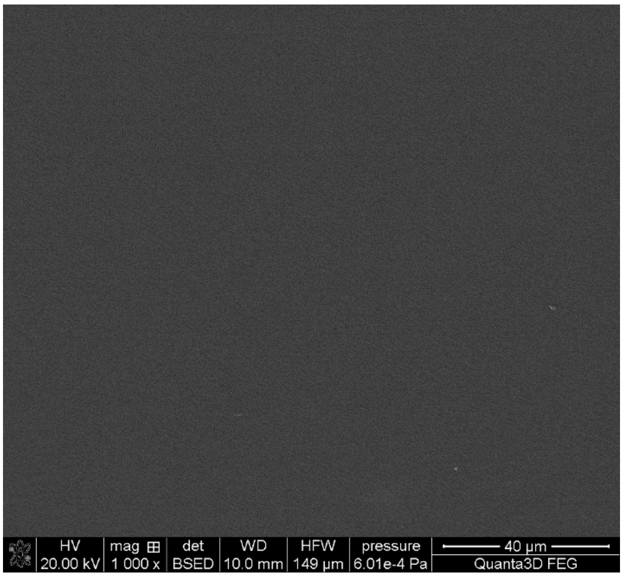

C

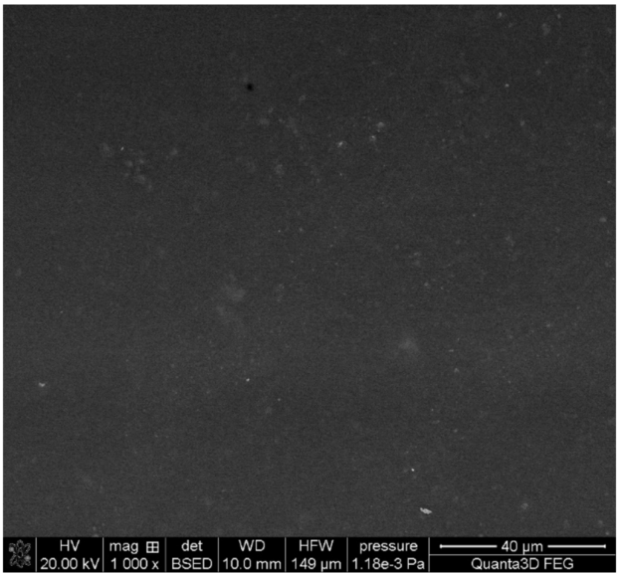

D

Figure S7. SEM photos of the studied materials: A: BPA.GDA-NVP0%; B: BPA.GDA-NVP3%; C: BPA.GDA-TMVS0%; D: BPA.GDA-TMVS3%.
